# Heat stress reduces the contribution of diazotrophs to coral holobiont nitrogen cycling

**DOI:** 10.1038/s41396-021-01158-8

**Published:** 2021-12-02

**Authors:** Nils Rädecker, Claudia Pogoreutz, Hagen M. Gegner, Anny Cárdenas, Gabriela Perna, Laura Geißler, Florian Roth, Jeremy Bougoure, Paul Guagliardo, Ulrich Struck, Christian Wild, Mathieu Pernice, Jean-Baptiste Raina, Anders Meibom, Christian R. Voolstra

**Affiliations:** 1 Red Sea Research Center, Division of Biological and Environmental Science and Engineering, King Abdullah University of Science and Technology, Thuwal, Saudi Arabia; 2 Department of Biology, University of Konstanz, Konstanz, Germany; 3 Laboratory for Biological Geochemistry, School of Architecture, Civil and Environmental Engineering, École Polytechnique Fédérale de Lausanne, Lausanne, Switzerland; 4 Metabolomics Core Technology Platform, Centre for Organismal Studies, University of Heidelberg, Heidelberg, Germany; 5 Baltic Sea Centre, Stockholm University, Stockholm, Sweden; 6 Faculty of Biological and Environmental Sciences, Tvärminne Zoological Station, University of Helsinki, Helsinki, Finland; 7 Centre for Microscopy, Characterisation and Analysis, University of Western Australia, Perth, WA Australia; 8 Museum für Naturkunde, Leibniz Institute for Evolution and Biodiversity Science, Berlin, Germany; 9 Department of Earth Sciences, Freie Universität Berlin, Berlin, Germany; 10 Faculty of Biology and Chemistry, Marine Ecology Department, University of Bremen, Bremen, Germany; 11 Climate Change Cluster, University of Technology Sydney, Sydney, NSW Australia; 12 Center for Advanced Surface Analysis, Institute of Earth Sciences, Université de Lausanne, Lausanne, Switzerland

**Keywords:** Microbial ecology, Climate-change ecology

## Abstract

Efficient nutrient cycling in the coral-algal symbiosis requires constant but limited nitrogen availability. Coral-associated diazotrophs, i.e., prokaryotes capable of fixing dinitrogen, may thus support productivity in a stable coral-algal symbiosis but could contribute to its breakdown when overstimulated. However, the effects of environmental conditions on diazotroph communities and their interaction with other members of the coral holobiont remain poorly understood. Here we assessed the effects of heat stress on diazotroph diversity and their contribution to holobiont nutrient cycling in the reef-building coral *Stylophora pistillata* from the central Red Sea. In a stable symbiotic state, we found that nitrogen fixation by coral-associated diazotrophs constitutes a source of nitrogen to the algal symbionts. Heat stress caused an increase in nitrogen fixation concomitant with a change in diazotroph communities. Yet, this additional fixed nitrogen was not assimilated by the coral tissue or the algal symbionts. We conclude that although diazotrophs may support coral holobiont functioning under low nitrogen availability, altered nutrient cycling during heat stress abates the dependence of the coral host and its algal symbionts on diazotroph-derived nitrogen. Consequently, the role of nitrogen fixation in the coral holobiont is strongly dependent on its nutritional status and varies dynamically with environmental conditions.

## Introduction

The association with microbial symbionts is central to the ecological success of reef-building corals in the oligotrophic tropical ocean [1, 2]. The close metabolic coupling between heterotrophic corals and their phototrophic algal symbionts supports their immense productivity, has given rise to their rapid radiation, and has led to the formation of coral reef ecosystems [3–7]. Consequently, the destabilization of this symbiosis in times of anthropogenic environmental change is posing a direct threat to the functioning of coral holobionts, i.e., the ecological unit of corals and their associated microorganisms, and the reefs they support [8–10]. In recent years, ocean warming has repeatedly caused mass coral bleaching, the breakdown of the coral-algal symbiosis, frequently followed by the death of the coral host and subsequent ecosystem-wide reef degradation [11, 12]. An in-depth understanding of the processes underlying the functioning and destabilization of the coral-algal symbiosis is thus required to predict or mitigate the effects of climate change on coral reefs [13–16].

Efficient recycling of organic and inorganic carbon in the coral-algal symbiosis depends on the nutrient-limited state of the algal symbionts [17–19]. In a stable state of the symbiosis, low bioavailable nitrogen availability limits algal growth and results in the accumulation of photosynthates in algal cells [20, 21]. The release of these excess nutrients, in turn, fuels the energy metabolism of the coral host. Although low levels of nitrogen assimilation are required to support the holobiont’s net productivity and growth [22], increases in the coral host’s catabolic activity during heat stress or other environmental stressors may increase nitrogen availability for the algal symbionts. Excess nitrogen availability may destabilize nutrient recycling in the coral-algal symbiosis resulting in a breakdown of coral holobiont functioning and eventually host starvation [15, 23–26]. The stability and functioning of the coral holobiont under changing environmental conditions thus depend on its ability to maintain a limited nitrogen availability for the algal symbionts [16, 25].

Importantly, nitrogen assimilation in the coral holobiont is not limited to the coral host and its algal symbionts. In addition to heterotrophic feeding and the uptake of nutrients from seawater, most coral holobionts also show detectable rates of nitrogen fixation, i.e., the prokaryotic conversion of atmospheric dinitrogen into bioavailable ammonium [27–29]. Diazotrophs, Bacteria, and Archaea, capable of nitrogen fixation, are common coral associates and show indications of host specificity [30–34]. As the abundance and activity of diazotrophs are highly dynamic and influenced by environmental change [35–39], nitrogen fixation may either stabilize or destabilize coral holobiont functioning depending on prevailing environmental conditions [25]. During periods of low environmental nitrogen availability, nitrogen fixation activity is positively correlated with coral holobiont productivity and could fulfill up to 11% of the algal symbionts’ nitrogen requirement [38]. In this context, the stimulation of nitrogen fixation rates by temperature stress or eutrophication has been proposed to enhance the productivity and resilience of coral holobionts during stress [40–42]. In addition, stimulated nitrogen fixation and increased nitrogen to phosphorus ratios in the holobiont have been reported during the breakdown of the coral-algal symbiosis under elevated sugar concentrations [43]. The importance of diazotrophs, thus, seems to depend on the fate of fixed nitrogen in the coral holobiont and its effects on other holobiont members [44]. However, our understanding of the environmental drivers controlling nitrogen fixation and the assimilation of diazotroph-derived nitrogen in the coral holobiont remains largely speculative at this point.

Recent studies showed that increased metabolic energy demands and the enhanced release of catabolic waste products may severely alter coral holobiont nutrient cycling during heat stress [15, 45]. We hypothesized that under such conditions, stimulated nitrogen fixation activity would enhance nitrogen availability for algal symbionts and thus contribute further to the destabilization of the coral-algal symbiosis. To test this, here we assessed the effects of heat stress on nitrogen fixation in the coral *Stylophora pistillata* from the central Red Sea. Combining amplicon sequencing, nutrient flux incubations, isotope labeling, and NanoSIMS imaging, we aimed to assess how heat stress affects diazotroph community structure, nitrogen fixation activity, and the assimilation of diazotroph-derived nitrogen in the coral holobiont before the onset of bleaching.

## Materials and methods

### Coral collection and experimental design

Five colonies of *S. pistillata* were collected at Abu Shosha reef (22°18ʹ16.3ʺN; 39°02ʹ57.7ʺE) in the central Red Sea close to the Saudi Arabian coast in September 2018. The colonies were collected at a water depth of ~5 m, at least 10 m apart, and were immediately transported to the indoor aquaria facilities of the Coastal and Marine Resources core laboratory (22°18′20.5″N; 39°06′ 14.3″E) at the King Abdullah University of Science and Technology (KAUST). The experiments for this study were conducted in parallel to those detailed in Rädecker et al. [15] using fragments of the same coral colonies and experimental setup. Coral colonies were fragmented into individual branches of ~5 cm length and nubbins were distributed over two 150 L aquaria per colony (5 colonies × 2 aquaria = 10 aquaria total). Each tank was filled with freshly collected seawater from Abu Shosha reef (salinity = 40.1 ± 0.2; NH_4_^+^ = 0.48 ± 0.03 μM; NO_3_^−^ = 0.19 ± 0.05 μM; PO_4_^3−^ = 0.03 ± 0.00 μM) with a daily water renewal rate of 25%, to maintain stable water parameters inside the aquaria over the course of the experiment (means ± SE for day 10 across aquaria and treatments: salinity = 40.1 ± 0.2; NH_4_^+^ = 0.48 ± 0.03 μM; NO_3_^−^ = 0.19 ± 0.05 μM; PO_4_^3−^ = 0.03 ± 0.00 μM). Seawater nutrient concentrations were measured in three technical replicates per sample using a SA3000/5000 nutrient auto-analyzer (Skalar Analytical B.V, Breda, The Netherlands) according to the manufacturer’s instructions. Each aquarium was equipped with a temperature controller (D-D The Aquarium Solution Ltd, Essex, UK), a 600-W heater (Schego, Offenbach, Germany), a current pump (Tunze, Penzberg, Germany), and a Radion light system (Ecotech Marine, Inc., Bethlehem, PA, USA) to maintain aquaria at a stable temperature of 29.1 °C and a light/dark regime mimicking in situ conditions of the collection site and depth (mean daytime irradiation = 380 μmol quanta m^−2^ s^−1^, peak daytime irradiation = 750 μmol quanta m^−2^ s^−1^). No supplemental feeding beyond naturally occurring seawater plankton was used in the experiment.

Following 7 days of acclimation, five of the aquaria (one per coral colony) were gradually ramped up to a temperature of 32.9 °C (absolute annual maximum at the collection site in 2017) over the course of 3 days, while the remaining five aquaria were maintained at a constant 29.1 °C (annual mean at the collection site in 2017) [46]. After 7 days at the maximum temperature (i.e., on day 10 of the experiment), replicate fragments from all colonies were sampled in both treatments for all molecular and physiological analyses. For molecular analyses, one nubbin per colony and treatment was immediately flash-frozen in liquid nitrogen and stored at −80 °C until further processing. For physiological analyses, one nubbin per colony and treatment was used for each of the incubations, which were performed immediately after the collection. Investigated response parameters included overall bacterial community composition (16S rRNA gene amplicon sequencing), diazotroph community composition (*nifH* amplicon sequencing), nitrogen fixation activity (acetylene reduction assay), net assimilation of diazotroph-derived nitrogen (^15^N_2_ isotope labeling + bulk analysis), and partitioning of diazotroph-derived nitrogen in the coral holobiont (NanoSIMS imaging of ^15^N_2_ isotope assimilation). For details on individual response parameters, see below.

### DNA extraction, amplification, and sequencing

Individual coral fragments were placed into Ziploc bags, covered in 0.6 mL of AP1 buffer (Qiagen, Hilden, Germany) and air-blasted to remove their tissue from the coral skeleton using air pressure for 1 min. RNAse A solution (6 µL from a 100 mg/mL stock) was immediately added to the retrieved tissue slurry in AP1 and 400 µL of the resulting slurry was directly used for DNA extraction with the DNeasy Plant Mini Kit (Qiagen) according to the manufacturer’s instructions. In parallel, 400 µL of AP1 buffer + RNase A were used for a no sample (null) DNA extraction to control for possible kit/lab contaminants. The yield and quality of all extractions were assessed using a Nanodrop 2000 (ThermoFischer, Waltham, MA, USA) [47].

For the characterization of the overall prokaryotic community composition, the hypervariable regions V5 and V6 of the 16S rRNA gene were amplified using the primers 784F 5′-TCGTCGGCAGCGTCAGATGTGTATAAGAGACAGAGGATTAGATACCCTGGTA-3′ and 1061R 5′-GTCTCGTGGGCTCGGAGATGTGTATAAGAGACAGCRRCACGAGCTGACGAC-3′ (Illumina overhang adaptor sequences are underlined) [47, 48]. PCRs were run in triplicate using a reaction volume of 10 µL with 5 µL of Qiagen multiplex PCR master mix, 10 ng of DNA template, and final primer concentrations of 0.5 µM for each primer, respectively. PCR cycling conditions consisted of an initial activation step at 95 °C for 15 min, followed by 27 cycles of 94 °C for 30 s, 55 °C for 30 s, and 72 °C for 30 s, followed by a final extension step at 72 °C for 10 min. Successful amplification of all samples was confirmed by gel electrophoresis before products of triplicate PCRs for each sample were pooled. PCR products were subjected to an indexing PCR (8 cycles) using the Nextera XT Index Kit v2 (Illumina, San Diego, USA) according to the manufacturer’s instructions. Indexed samples were then cleaned and normalized using the SequalPrep Normalization Plate Kit (Invitrogen, Carlsbad, CA, USA), pooled at equimolar ratios, and verified on the Agilent 2100 Bioanalyzer (Agilent Technologies, Santa Clara, CA, USA). Amplicon libraries were sequenced at the KAUST Bioscience core laboratory on the MiSeq platform (Illumina) using 2 × 301 bp overlapping paired-end reads with 20% spiked-in phiX.

For the characterization of the diazotroph community composition, the primers IGK3 5′-TCGTCGGCAGCGTCAGATGTGTATAAGAGACAGGCIWTHTAYGGIAARGGIGGIATHGGIAA-3′ and DVV 5′-GTCTCGTGGGCTCGGAGATGTGTATAAGAGACAGATIGCRAAICCICCRCAIACIACRTC-3′ (Illumina overhang adaptor sequences are underlined) were used to amplify a region of the *nifH* gene due to their broad taxonomic range and high specificity as discussed in Gaby and Buckley [49]. PCRs were run in triplicate using a reaction volume of 10 µL with 5 µL of Qiagen multiplex PCR master mix, 10 ng of DNA template, and final concentrations of 1.2 µM for each primer, respectively. PCR cycling conditions consisted of an initial activation step at 95 °C for 15 min, followed by 40 cycles of 95 °C for 45 s, 57 °C for 45 s, and 72 °C for 60 s, followed by a final extension step at 72 °C for 10 min. Successful amplification of all samples was confirmed by gel electrophoresis before products of triplicate PCRs for each sample were pooled and purified using Agencourt AMPure beads (Agencourt Bioscience Corporation, Beverly, MA, USA). Purified PCR products were subjected to an indexing PCR (8 cycles) using the Nextera XT Index Kit v2 (Illumina, San Diego, CA, USA) according to the manufacturer’s instructions. Indexed samples were then cleaned and normalized using the SequalPrep Normalization Plate Kit (Invitrogen), pooled at equimolar ratios and verified on the Agilent 2100 Bioanalyzer (Agilent Technologies). Amplicon libraries were sequenced at the KAUST Bioscience core laboratory on the MiSeq platform (Illumina) using 2 × 301 bp overlapping paired-end reads with 20% spiked-in phiX.

### Sequence processing and analysis

For the 16S rRNA gene-based characterization of the prokaryotic community composition, demultiplexed sequence reads (~54,000 per sample on average) were processed in “DADA2” v.1.18.0 [50] running in “R” v.4.0.3 [51]. Following removal of primer sequences in “DADA2,” reads were quality filtered with a maximum expected error (max EE) of 2, poor quality read ends were trimmed (truncQ = 2), and forward and reverse reads were truncated at 240 and 160 bp, respectively. The error rates were estimated based on all samples and used for inference of true sequence variants. Following the merging of paired reads and removal of chimeras, amplicon sequence variants (ASVs) were classified against the SILVA database version 138 [52] and all non-bacterial ASVs, as well as those with more than 1% relative abundance in negative extractions, were removed from the dataset in “phyloseq” v.1.34.0 [53]. This yielded an average of ~35,000 retained reads per sample distributed over 1,797 ASVs. The effect of the temperature treatment on the bacterial community composition was assessed at the ASV level. For this, the dataset was cleaned from sparsely distributed ASVs using “pime” v.0.1.0 with a prevalence threshold of 40% (~23,000 reads per sample after filtering) and communities were compared using analysis of similarities (ANOSIM) with Bray–Curtis dissimilarities calculated from relative abundances of ASVs as implemented in “vegan” 2.5-7 [54, 55]. Differential abundance of bacterial taxa between temperature treatments was assessed using linear discriminant analysis effect size (LEfSe) as implemented in the “microbial” R package v.0.0.19 [56].

For the *nifH*-based characterization of the diazotroph community composition, primers were removed from demultiplexed sequence reads (~202,000 per sample) with “Cutadapt” v.2.10 [57]. Using “DADA2” v.1.18.0 [50] running in “R” v.4.0.3 [51], reads were quality filtered with a max EE of 2, poor quality read ends were trimmed (truncQ = 2), and forward and reverse reads were truncated at 220 and 160 bp, respectively. The error rates were estimated based on all samples and used for inference of true sequence variants. Following the merging of paired reads and removal of chimeras, ASVs were translated to the protein level with FrameBot v.1.2.0 using the included *nifH* reference file [58]. Translated sequences were assigned to *nifH* gene clusters following Meunier et al. [59], using a consensus assignment based on three criteria adapted after Angel et al. [60]: first, sequences were aligned in “MAFFT” v7.475 [61] and analyzed using a Classification and Regression Tree model following Frank et al. [62]. Second, an Evolutionary Placement Algorithm tree [63] was constructed using the corresponding RaxML implementation and sequences, and were placed on the reference tree after alignment against a reference dataset containing all *nifH* gene clusters, as implemented in the NifMAP pipeline [60]. Third, sequences were queried against the curated *nifH* gene database provided by the Zehr lab (https://wwwzehr.pmc.ucsc.edu/nifH_Database_Public) for gene clustering as well as taxonomic assignment. Based on this approach, ASVs assigned to *nifH* paralogs not involved in nitrogen fixation (clusters IV&V) as well as all ASVs with more than 1% relative abundance in negative extraction were excluded from the dataset in “phyloseq” v.1.34.0 [53]. This yielded an average of ~65,000 retained reads per sample (93,000–186,000 reads including *nifH* paralogs) distributed over 238 ASVs (876 ASVs including *nifH* paralogs). The effect of the temperature treatment on the diazotroph community composition was assessed at the ASV level. To do this, the dataset was cleaned from sparsely distributed ASVs using “pime” v.0.1.0 with a prevalence threshold of 60% (~42,000 reads per sample after filtering) and communities were compared using ANOSIM with Bray–Curtis dissimilarities calculated from relative abundances of ASVs as implemented in “vegan” 2.5-7 [46, 47]. Differential abundance of diazotroph taxa between temperature treatments was assessed using LEfSe as implemented in the “microbial” R package v.0.0.19 [56].

### Acetylene reduction assays

Nitrogen fixation rates of coral holobionts were quantified indirectly using the acetylene (C_2_H_2_) reduction assay [64]. For this, corals were transferred into 1 L glass incubation chambers (Weck, Wehr-Öflingen, Germany) and submerged in filtered seawater (0.22 µm) to a final volume of 720 mL. After this, 80 mL of C_2_H_2_-saturated seawater (continuously bubbled with C_2_H_2_ for 30 min prior to incubations) was added and the chambers were sealed gas-tight with a modified glass lid containing a syringe injection port. Ten percent (20 mL) of the gas headspace was replaced with pure acetylene. Incubation chambers were transferred into temperature-regulated water baths equipped with magnetic stirrers and Radion lights (Ecotech Marine, Inc.) to maintain temperature and light conditions identical to treatment and aquaria conditions. At the beginning and after 24 h of incubation, 2.5 ml gas samples were collected from the chamber headspace with a gas-tight syringe equipped with a push-pull valve (Trajan, Ringwood, Australia) and transferred into vacuum blood collection tubes for subsequent analysis. Ethylene (C_2_H_4_) concentrations in the gas samples were determined using gas chromatography with a helium pulsed discharge detector (Agilent 7890B GC system with HP-Plot/Q column; lower detection limit for C_2_H_4_: 0.3 p.p.m.). Rates of C_2_H_4_ evolution during the incubations were calculated based on changes in C_2_H_4_ concentrations in the chamber taking into account the temperature-dependent solubility of C_2_H_4_ in seawater [65] as well as background fluxes of seawater controls without corals to account for possible planktonic background C_2_H_4_ production (ca. an order of magnitude lower than in coral incubations) [65]. C_2_H_4_ evolution rates were converted to their equivalent nitrogen fixation rates using the theoretical conversion factor of 4 and normalized to the surface area of coral fragments [66]. Importantly, the choice of the correct conversion factor is widely debated and may depend on the species and environmental context, as such absolute rates should be interpreted with caution [67]. For rate normalization, surface areas of coral fragments were estimated by 3D computer modeling [68]. Forty to 50 photos were taken of the coral fragments from all angles. The Autodesk Photo-to-3D cloud service (Autodesk, Mill Valley, CA, USA) was used to generate 3D models, which were used for surface area analysis with ReCap Photo v.4.2.0.2 (Autodesk).

### Isotope labeling and bulk isotope analysis

To quantify the assimilation of microbially fixed nitrogen in the coral holobiont, an isotope labeling approach with ^15^N_2_ isotope-enriched seawater was adapted after the dissolution technique outlined in Wilson et al. [69]. First, filtered seawater (0.22 µm) was degassed by constant stirring at low pressure (<200 mbar) for 30 min and degassed seawater was transferred into 1 L borosilicate bottles with gas-tight rubber septa. To allow for higher ^15^N_2_ concentrations during incubations without depleting oxygen (O_2_) levels, bottles were injected with 25 mL of a gas mixture containing 80% ^15^N_2_ (98 atom%, Cambridge Isotope Laboratories, Tewksbury, MA, USA) and 20% O_2_ using a gas-tight syringe equipped with a push-pull valve (Trajan). Bottles were vigorously shaken until the bubble volume remained stable and the bottles were stored under constant agitation at 4 °C overnight. ^15^N_2_-enriched seawater and freshly filtered seawater (0.22 µm) were mixed at equal parts for the final incubations. Notably, this approach did not compensate for the reduction of pCO_2_ during the degassing. As such, we cannot rule out that alterations in seawater carbonate chemistry (albeit consistent across all treatments) may have had minor effects on coral physiology during the incubations. For the isotope labeling, corals were incubated in bubble-free, sealed 1 L glass chambers (Weck) with gas-tight glass lids. Incubation chambers were transferred into temperature-regulated water baths equipped with magnetic stirrers and Radion lights (Ecotech Marine, Inc.) to maintain temperature and light conditions identical to treatment and aquaria conditions. After 24 h, the seawater in the incubations was replaced with equal parts of ^15^N_2_-enriched seawater and freshly filtered seawater (0.22 µm), and the chambers were sealed again for another 24 h of incubation. After a total of 48 h, incubations were terminated and corals were sampled for isotope analysis. The tip of coral fragments (~0.5 cm) was removed using a clipper and processed for NanoSIMS analyses as outlined below. The remaining fragment was transferred into a ziplock bag, covered in 2 mL of deionized water, and its tissue was removed from the coral skeleton using air pressure. The resulting tissue slurry was freeze-dried and homogenized subsamples were transferred into tin capsules for bulk N isotope analysis. Total nitrogen content, as well as ^15^N/^14^N ratios, were measured using an elemental analyzer (Thermo Flash EA 1112) coupled to a stable isotope mass spectrometer (IRMS, DELTA V Advantage). Notably, we did not quantify concentrations of ^15^N_2_ in the incubation water. Hence, ^15^N/^14^N ratios do not allow for absolute quantification of nitrogen assimilation rates in the present study. However, as all corals were incubated in the same incubation water with identical ^15^N_2_ concentrations, levels of ^15^N enrichments provide a direct reflection of relative nitrogen assimilation rates in these corals. For this, levels of ^15^N enrichment were expressed using the delta (δ) notation in units per mil of stable isotope ratios calculated as: δ^15^N (‰) = (^15^N/^14^N_labeled sample_/^15^N/^14^N_unlabeled sample_ − 1) × 1,000.

### NanoSIMS imaging

Following ^15^N_2_ incubations, tips of coral fragments were immediately transferred into a fixative solution (1.25% glutaraldehyde and 0.5% paraformaldehyde in 0.1 M phosphate buffer) at 4 °C for 24 h. Fixed samples were washed once in phosphate-buffered saline (1×), decalcified in 0.1 M ethylenediaminetetraacetic acid (exchanged daily) for 2 weeks after which the tissue was dissected into a strip of two to three individual polyps. The tissue was dehydrated in a series of increasing ethanol concentrations (50%, 70%, 90%, and 100%), transferred to acetone, and gradually infiltrated with increasing concentrations of SPURR resin (25%, 50%, 75%, and 100%). Subsequently, tissues were embedded in SPURR resin and cut into 170 nm sections using an Ultracut E microtome (Leica Microsystems, Wetzlar, Germany) and mounted on pulse-discharge silicon wafers. Wafers with sample sections were gold-coated (ca. 15 nm) and analyzed with the NanoSIMS 50 ion probe [70] at the Center for Microscopy, Characterization, and Analysis at the University of Western Australia. Surfaces of samples were bombarded with a 16 keV primary Cs^+^ beam focused to a spot size of about 100 nm with a current of ~2 pA. Secondary molecular ions ^12^C^12^C^−^, ^12^C^13^C^−^, ^12^C^14^N^−^, and ^12^C^15^N^−^ were simultaneously collected in electron multipliers at a mass resolution (*M*/Δ*M*) of about 8,000. Images for all targeted secondary molecular ions were collected by rastering the primary beam across the sample with a dwell time of 9 ms per pixel; six planes were recorded for each area. Charge compensation was not necessary. Although samples from all coral colonies were analyzed on the NanoSIMS, only data and images from the colony with the highest ^15^N enrichment level in its tissue (including both control and heat stress conditions) are shown here. For this colony, at least 25 images across the polyp tissue (30 μm raster with 256 × 256 pixels) were collected from both treatments, respectively. To avoid any biases between samples due to differences in analyzed tissue areas within the polyp, only images containing both host tissue and algal symbiont cells were included in the analysis. Following this criterion, 15 images from the control as well as the heat treatment were used for the comparison of ^15^N/^14^N ratios, respectively. Images were processed with the ImageJ plugin OpenMIMS (National Resource for Imaging Mass Spectrometry, https://github.com/BWHCNI/OpenMIMS/wiki). After drift correction, the individual planes were summed and the ^12^C^14^N^−^ maps were used to draw two regions of interest (ROI) per image containing either all host gastrodermis or all algal symbionts cells and the δ^15^N of each ROI was calculated as outlined above.

### Statistical analyses of nitrogen fixation activity and assimilation rates

All statistical analyses were performed in “R” v.4.0.3 [51]. Differences in nitrogen fixation rates (C_2_H_2_ reduction), as well as ^15^N_2_ assimilation (IRMS), were analyzed in a paired design based on colony replicates using a sign test. The relationship between nitrogen fixation rates and ^15^N_2_ assimilation was assessed via a correlation analysis using Pearson’s product–moment correlation coefficient. NanoSIMS measurements of δ^15^N in the host and symbiont tissue/cells were analyzed using a two-way analysis of variance using holobiont compartment and treatment as explanatory variables.

## Results and discussion

### Diazotroph community composition varies with temperature

To assess the role of diazotrophs in the early response of the coral holobiont to heat stress, we sampled the colonies of *S. pistillata* on day 10 of the experiment (7 days at a maximum temperature of 32.9 °C). At this time point, corals from heat stress and control treatments maintained a healthy appearance, similar levels of algal symbiont densities, and showed no visual signs of bleaching (for an extended discussion of processes and timeframes of bleaching during the experiment, please refer to Rädecker et al. [15]). The early heat-stress response did not affect overall bacterial community composition (ANOSIM, *p* = 0.145; Fig. S1). Members of the order Oceanospirillales, which are prevalent in healthy corals and *S. pistillata* in particular [48, 71, 72], represented the largest component of the coral microbiomes from both treatments (~20% of 16S rRNA sequences).

**Fig. 1:**
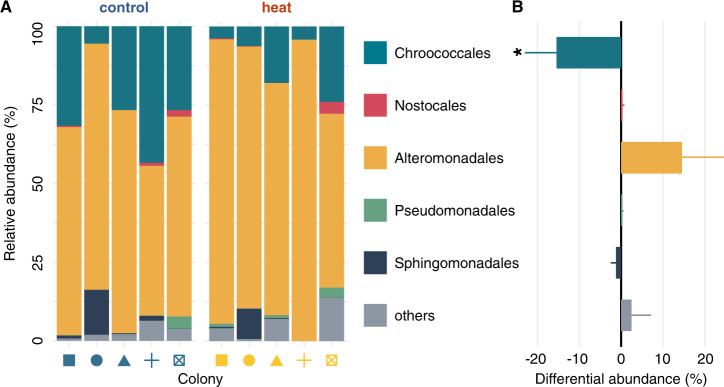
Characterization of coral-associated diazotroph communities after 10 days of heat stress. **A** Relative diazotroph community composition of individual colonies under control (left) and heat stress (right) conditions based on *nifH* amplicon sequencing. Notably, no archaeal diazotrophs could be detected in the present study. **B** Change in the relative abundance of dominant diazotroph orders during heat stress relative to control conditions (mean ± SE). Asterisks indicate a significant change (*p* < 0.05) under heat stress compared to control corals.

In contrast to the stable overall bacterial microbiome composition, *nifH* sequencing revealed that heat stress caused a distinct shift in the community composition of diazotrophs (ANOSIM, *p* = 0.049). This shift was not driven by the loss or the recruitment of novel taxa but rather by variations in the relative abundance of taxa already present in the holobiont (Fig. 1A, B). The diazotroph community was largely dominated by the orders Alteromonadales and Chroococcales accounting for 65% and 27% of *nifH* sequences in colonies from the ambient control, respectively. Heat stress, however, caused a significant decline in the relative abundance of Chroococcales (LEfSe, *χ*^2^ = 3.94, *p* = 0.047) accompanied by the proportional (albeit not significant) increase of Alteromonadales in the diazotroph community (LEfSe, *χ*^2^ = 2.46, *p* = 0.117) (Fig. 1B). Importantly, Chroococcales were previously identified as endosymbionts in the epithelium of the Caribbean coral *Montastrea cavernosa* [73] and have been proposed to be an important source of fixed nitrogen for algal symbionts in these corals [74]. Consequently, the observed shifts in diazotroph community composition could directly affect nitrogen availability for other holobiont members during heat stress.

### Algal symbionts assimilate diazotroph-derived nitrogen in a stable coral holobiont

**Fig. 2:**
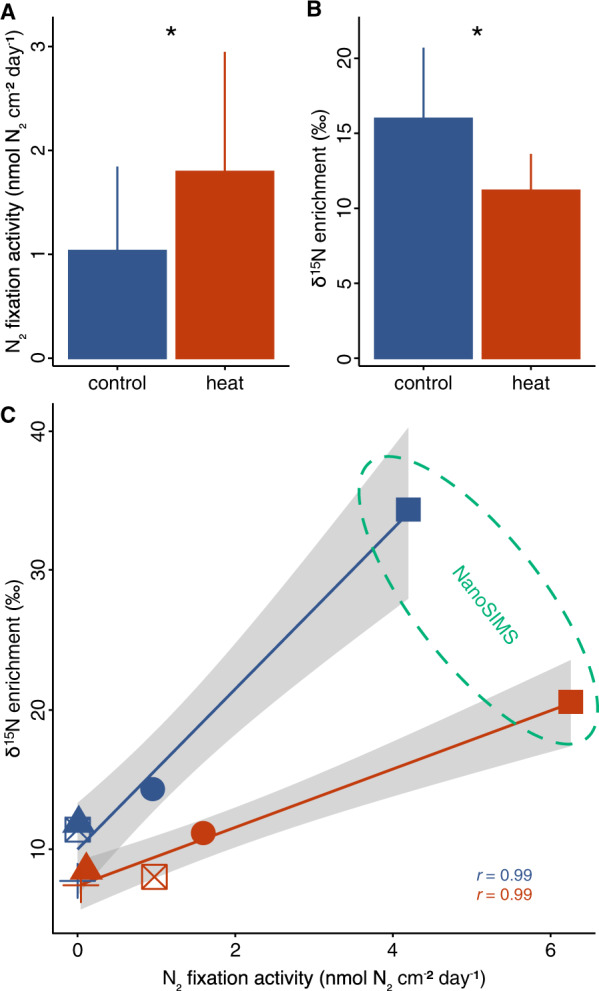
Nitrogen fixation activity and assimilation in the coral holobiont after 10 days of heat stress. **A** Nitrogen fixation activity of coral holobionts (*n* = 5 per treatment) was quantified via the acetylene reduction assay. **B** Net assimilation of diazotroph-derived nitrogen in the coral holobiont (*n* = 5 per treatment) quantified by ^15^N_2_ isotope labeling and bulk stable isotope analysis. **C** Nitrogen fixation activity and assimilation of diazotroph-derived nitrogen show a positive correlation across colonies (symbols) under control (blue) and heat stress conditions (red) with corresponding confidence intervals (gray). Pearson’s correlation coefficients (*r*) correspond to correlations among each condition of the corresponding color. Samples from both control and heat stress conditions of the colony with the highest enrichment (ellipse) were imaged in detail using the NanoSIMS ion microprobe. Bars and error bars indicate mean ± SE. Asterisks indicate significant differences between treatments (*p* < 0.05).

Using the acetylene reduction technique, we estimated daily nitrogen fixation rates in *S. pistillata* holobionts. Under ambient control conditions (i.e., 29.1 °C), coral holobionts showed detectable rates of nitrogen fixation around 1.0 ± 0.8 nmol N_2_ cm^−2^ day^−1^ in line with rates previously reported for corals from this region (Fig. 2A) [31, 75, 76]. Further, ^15^N_2_ isotope labeling resulted in enriched δ^15^N values in the soft tissue of coral holobionts (mean ± SE = 16.0 ± 4.7‰), which positively correlated with nitrogen fixation rates across coral colonies (Pearson’s correlation, *r* = 0.99, *p* = 0.002, Fig. 2B, C). Although these nitrogen fixation rates may be relatively low compared to other nutrient sources, they are a non-negligible component of the overall nutrient cycling in the nitrogen-limited *S*. *pistillata* holobiont.

**Fig. 3:**
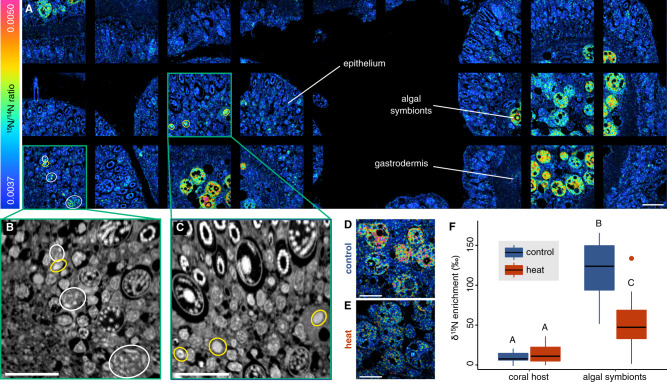
NanoSIMS imaging of ^15^N_2_ assimilation in the coral holobiont after 10 days of heat stress. **A** Mosaic of hue saturation images of ^15^N^12^C^−^/^14^N^12^C^−^ ratios illustrating the spatial distribution of ^15^N enrichment in the coral tissue under control conditions. **B**, **C** Enlarged ^14^N^12^C^−^ NanoSIMS images of epithelial ^15^N_2_ hotspots highlighted in **A**. Epithelial hotspots were primarily characterized by two distinct morphologies: oval compartments/cells with 2–3 µm length (yellow circles) and clusters of smaller rod-shaped compartments/cells with 1–3 µm in length (white circles). **D**, **E** Hue saturation images of ^15^N^12^C^−^/^14^N^12^C^−^ ratios illustrating ^15^N assimilation in the coral host and algal symbionts under control (**D**) and heat stress (**E**) conditions. **F** ^15^N_2_ assimilation in the coral tissue and algal symbionts under control (blue) and heat stress (red) conditions based on NanoSIMS images (*n* = 15 per treatment). Differing letters above the boxplot indicate significant differences between groups (*p* < 0.05). All scale bars are 10 µm.

**Fig. 4:**
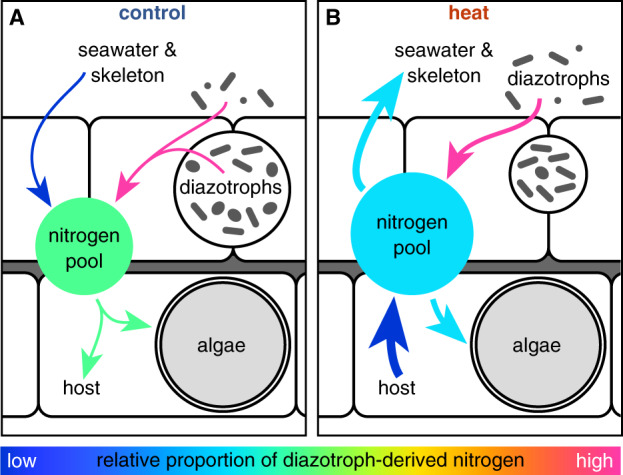
Conceptual view of the effect of heat stress on nitrogen assimilation in the coral holobiont. **A** In a stable state, low environmental nitrogen availability limits nitrogen uptake in the coral holobiont. Under these conditions, diazotroph-derived nitrogen provides an important nitrogen source for the coral-algal symbiosis. **B** During heat stress, the release of excess nitrogen waste from the host metabolism increases nitrogen availability in the coral holobiont. Under these conditions, the coral-algal symbiosis does not depend on diazotroph-derived nitrogen. The color of arrows indicates the proportion of diazotroph-derived nitrogen in the nitrogen flux/pool: continuous scale ranging from “low contribution of diazotroph-derived nitrogen (dark blue)” on one end of the spectrum to “high contribution of diazotroph-derived nitrogen (pink)” on the other end of the spectrum. The line width of arrows in the heat stress (**B**) indicates their proportional increase relative to control conditions (**A**).

To identify which holobiont partner(s) assimilated diazotroph-derived nitrogen in the coral holobiont, we used NanoSIMS imaging to trace the incorporation of the ^15^N_2_ isotope marker in the coral colony with the highest nitrogen fixation activity (Fig. 3A–D). Overall, the algal symbionts showed significantly higher levels of ^15^N enrichment than the coral host tissue (118.7 ± 9.4‰ (symbionts), 9.5 ± 1.6‰ (host); Tukey’s honestly significant difference (HSD), *p* < 0.001, Fig. 3F). These observations are consistent with previous reports for *S. pistillata* from the Great Barrier Reef and show that a large fraction of diazotroph-derived nitrogen is eventually assimilated by the algal symbionts [33]. In addition, NanoSIMS images revealed the presence of small ^15^N enrichment hotspots in the host epithelium that displayed two distinct morphologies: oval compartments/cells 2–3 µm in length and clusters of smaller circular and rod-shaped compartments/cells with 1–3 µm in length (Fig. 3B, C). Although the NanoSIMS images do not allow us to elucidate the identity of these hotspots, their shape and location are broadly consistent with previous reports of the presence of endosymbiotic bacteria in the coral holobiont [73, 77–79]. Specifically, the larger oval hotspots resemble observations of Chroococcales in the epithelium of *M. cavernosa*, whereas the rod-shaped clusters resemble previously described bacterial aggregates in the coral tissue in their structure and localization [73, 77–79]. It is thus plausible that the observed epithelial ^15^N hotspots represent endosymbiotic diazotrophs in the tissue of *S. pistillata*. However, if these ^15^N hotspots are indeed diazotrophs, they are not situated near the algal symbionts that are the primary sink of diazotroph-derived nitrogen. In many other phototroph–diazotroph symbioses, diazotrophs predominantly release fixed nitrogen via the passive diffusion of ammonium [80]. Although the ways of nitrogen release by coral-associated diazotrophs are currently unknown, it is plausible that passive ammonium release and/or the catabolic breakdown of diazotrophs during host digestion may contribute to the inorganic nitrogen pool in the holobiont. If this is the case in *S. pistillata*, the uneven partitioning of diazotroph-derived nitrogen between the coral host and its algal symbionts likely reflects the respective metabolic demands of symbiotic partners for external nitrogen sources. In this scenario, the nitrogen-limited state of algal symbionts might support strong concentration gradients in the host tissue, which would enable efficient assimilation of exogenous nitrogen (derived from either diazotrophs or from the surrounding seawater) [81]. In a stable holobiont state, diazotrophs thus provide a nitrogen source to support algal symbiont growth and allow for net productivity of the coral holobiont (Fig. 4A).

### Reduced assimilation of fixed nitrogen by algal symbionts despite increased nitrogen fixation activity during heat stress

On day 10 of the experiment, heat stress caused a 74% increase in nitrogen fixation rates (indirectly quantified as acetylene reduction) compared to coral holobiont rates from ambient control conditions (sign test, *z* = 2.24, *p* = 0.025, Fig. 2A). Similar increases in coral-associated nitrogen fixation during heat stress have been previously reported for other coral species [40, 41], suggesting that the here reported processes are unlikely restricted to the *S. pistillata* holobiont, but may be a common feature of coral-associated diazotroph communities. ^15^N_2_ isotope labeling in this study revealed that the additional fixed nitrogen was not assimilated in the soft tissue of the holobiont, in which ^15^N enrichment decreased by 30% compared to ambient controls (sign test, *z* = 2.24, *p* = 0.025, Fig. 2B). NanoSIMS analysis corroborated this observation, revealing that the ^15^N enrichment remained stable in the host tissue (Tukey’s HSD, *p* = 0.976) but declined by 53% in the algal symbionts compared to ambient controls (Tukey’s HSD, *p* < 0.001, Fig. 3E, F). Furthermore, we did not detect any epithelial ^15^N enrichment hotspots in corals exposed to heat stress, suggesting lower abundances of diazotrophs and/or lower nitrogen fixation activity. The overall increase in nitrogen fixation activity in the coral holobiont during heat stress (Fig. 2A), hence, was likely not driven by endosymbiotic diazotrophs in the coral tissue and did not result in increased assimilation of diazotroph-derived nitrogen by the algal symbionts in the present study (Fig. 3F).

Importantly, the dependence of algal symbionts on diazotroph-derived nitrogen is a function of their nutritional status. In this context, Rädecker et al. [15] recently showed (using the same coral colonies and experimental design) that heat stress shifted these coral holobionts from a state of nitrogen limitation towards a state of carbon limitation. Specifically, energy starvation caused by heat stress promoted the catabolic generation of inorganic nutrients (including ammonium) in the coral tissue, thereby promoting the proliferation of algal symbionts (Fig. S2). This ammonium released by the host catabolism was not isotopically enriched and therefore diluted diazotroph-derived nitrogen in the inorganic nutrient pool. Overall, the reduced ^15^N enrichment observed here suggests that heat stress reduces the relative contribution of diazotroph-derived nitrogen to the coral holobiont nitrogen pool (Fig. 4B). In this scenario, altered nutrient cycling may help explain the absence of epithelial ^15^N enrichment hotspots during heat stress. Low photosynthate and high ammonium availability in the coral tissue likely suppress the nitrogen fixation activity of endosymbiotic diazotrophs and may give a competitive advantage to other microbes better adapted to exploit the altered nutrient regime during heat stress [82–84].

It is important to consider that the bulk isotope and NanoSIMS analyses only quantify the assimilation of anabolically incorporated ^15^N into the coral and symbiont cells. As such, the assimilation of diazotroph-derived nitrogen in other holobiont compartments (i.e., the coral skeleton and the surface mucus layer) is not accounted for in our analyses. Indeed, a study by Moynihan et al. [34] recently suggested that endolithic microbes in the coral skeleton were the main source and sink of diazotroph-derived nitrogen in the closely related *Pocillopora acuta* holobiont. Further, El-Khaled et al. [85] showed that moderate increases in inorganic nutrient concentrations may stimulate nitrogen fixation rates in Red Sea corals. In this context, the observed net release of ammonium and phosphate by the coral host during heat stress would directly affect nutrient availability in other compartments of the coral holobiont such as the skeleton (Fig. S2 [15]). It is thus plausible that the increase in nitrogen fixation activity described here was predominantly driven by an increase in the activity of endolithic microbes associated with the holobiont. While some studies suggested that endolithic microbial communities may eventually release some of their nutrients to the coral tissue [34, 42, 86, 87], our results clearly show that diazotroph-derived nitrogen (regardless of its origin within the holobiont) is an insignificant source of nitrogen for the coral-algal symbiosis during heat stress. As such, the increase in stimulated nitrogen fixation may primarily be absorbed within the endolithic community itself, thereby supporting the frequently documented rapid proliferation of endolithic microbes during heat stress [86, 88, 89]. Further, nitrogen fixation rates correlate with denitrification activities in some Red Sea corals [75]. Although we did not quantify denitrification rates in the present study, increases in the abundance and or activity of denitrifying microbes may have contributed to the removal of diazotroph-derived nitrogen from the coral holobiont during heat stress. Taken together, we conclude that the excess availability and release of ammonium through host catabolic processes, combined with increased microbial utilization of fixed nitrogen, likely reduce the relative contribution of diazotroph-derived nitrogen to holobiont nitrogen cycling during heat stress.

### The functional importance of diazotrophs in the coral holobiont

Nitrogen fixation rates in coral holobionts are highly variable—they can differ between host species, locations, and fluctuate depending on local environmental conditions [34, 37, 38, 41, 42, 82, 87, 90]. Indeed, the molecular, physiological, and ultrastructural characterization of nitrogen fixation presented here paints a complex picture of the role of diazotrophs in coral holobiont functioning. Our results suggest that endosymbiotic diazotrophs in the coral epithelium actively fix nitrogen in the coral holobiont. The characterization of tissue-associated diazotroph communities suggests that the observed ^15^N hotspots may resemble individual endosymbiotic diazotrophs of the order Chroococcales and bacterial aggregates potentially including diazotrophs of the order Alteromonadales. Further, the disappearance of these ^15^N hotspots during heat stress coincided with a decline in Chroococcales *nifH* relative sequence abundance and the reduced ^15^N assimilation in algal symbiont cells. Taken together, our findings indicate that endosymbiotic diazotrophs in the coral tissue, especially Chroococcales, represent an important source of nitrogen for algal symbionts under nitrogen-limited conditions. Hence, the association with diazotrophs may supplement holobiont nutrition and enable net productivity under oligotrophic conditions [38]. However, this beneficial role of diazotrophs is likely limited to a narrow window of environmental conditions in which algal symbionts are strongly nitrogen-limited. Environmental conditions that reduce the demand for nitrogen in the coral holobiont (e.g., heat stress in the present study), may hence undermine the beneficial role of diazotrophs in holobiont functioning. This dynamic dependence on diazotroph-derived nitrogen in the coral holobiont is directly reflected in the variable diazotroph community assemblage, their activity, and the fate of diazotroph-derived nitrogen in the coral holobiont and may indirectly facilitate holobiont acclimatization and adaptation to changing levels of nutrient availability. In other words, this study suggests that the nutritional status of the coral holobiont determines the structure and activity of associated diazotroph communities and not vice versa. As such, diazotrophs may support coral holobiont functioning under nitrogen-limited conditions. However, anthropogenic impacts such as eutrophication and ocean warming likely reduce the benefits of hosting diazotrophs in the coral holobiont.

## Supplementary information

Supplemental Material

## Data Availability

All raw sequencing data have been deposited under BioProject ID PRJNA741490 (https://www.ncbi.nlm.nih.gov/bioproject/PRJNA741490). Further, ASV abundance tables (16S rRNA and *nifH* gene amplicons) as well as physiological and NanoSIMS data have been deposited at Zenodo.org and are freely available at 10.5281/zenodo.5552980.
