# Supplementary material for: Heat stress reduces the contribution of diazotrophs to coral holobiont nitrogen cycling
Source: ISME J. 2021 Dec 2;16(4):1110–8. doi: 10.1038/s41396-021-01158-8 (PMC8941099; doi:10.1038/s41396-021-01158-8)
Supplement: Supplementary file 1 — Supplemental Material [file 41396_2021_1158_MOESM1_ESM.pdf]

Supplementary Materials

Heat stress reduces the contribution of diazotrophs to coral holobiont nitrogen cycling

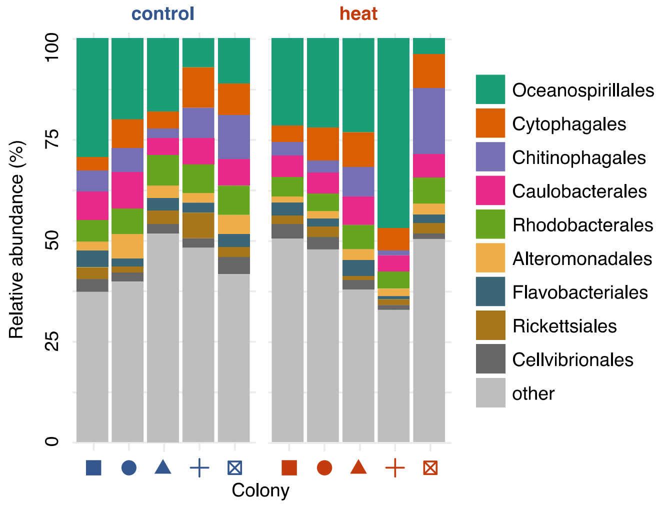

Figure S1. Coral-associated bacterial communities after 10 days of heat stress. Bars illustrate the relative abundance of 16S rRNA gene amplicon sequences of dominant bacterial orders from coral colonies from control (left) and heat stress (right) conditions. Symbols below bars indicate the five colonies used in the experiment.

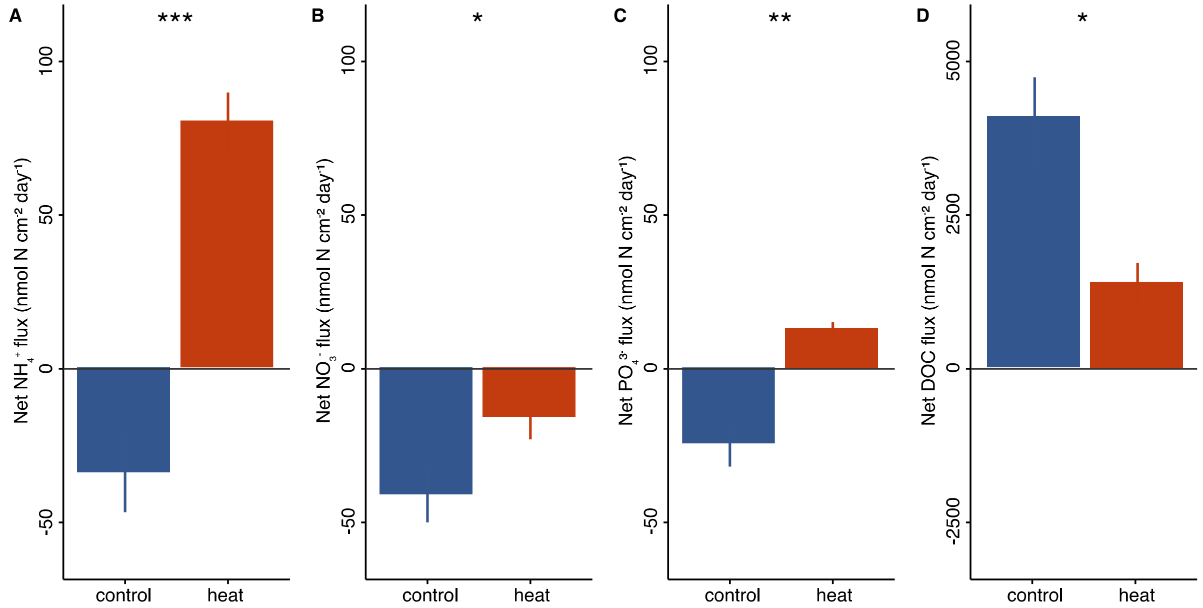

Figure S2. Net nutrient fluxes between coral holobionts and surrounding seawater during 24 h incubations at day 10 of heat stress. A Net ammonium (NH_4^+) fluxes. B Net nitrate (NO_3^-) fluxes. C Net phosphate (PO_4^{3-}) fluxes. D Net dissolved organic carbon (DOC) fluxes. All fluxes are normalized to the surface area of the coral fragment. Positive fluxes indicate a net release of nutrients by the coral holobiont into the surrounding seawater. Negative fluxes indicate a net uptake of nutrients by the coral holobiont from the surrounding seawater. Raw data for this plot taken from Rädecker et al. [1]. Please refer to Rädecker et al. [2] for further details on incubations and analyses. Asterisks indicate significant differences between heat stress and control treatments (* $p < 0.05$; ** $p < 0.01$, *** $p < 0.001$).

Supplementary references

1. Rädecker N, Pogoreutz C, Gegner HM, Cárdenas A, Roth F, Bougoure J, et al. Data for “Heat stress destabilizes symbiotic nutrient cycling in corals.” Zenodo.org 2021. https://doi.org/10.5281/zenodo.4429583

2. Rädecker N, Pogoreutz C, Gegner HM, Cárdenas A, Roth F, Bougoure J, et al. Heat stress destabilizes symbiotic nutrient cycling in corals. *Proc Natl Acad Sci U S A*. 2021; 118: e2022653118
